# Anti-Inflammatory Activity of N-Butanol Extract from *Ipomoea stolonifera In Vivo* and *In Vitro*

**DOI:** 10.1371/journal.pone.0095931

**Published:** 2014-04-21

**Authors:** Congyi Cai, Yicun Chen, Shuping Zhong, Bin Ji, Jinzhi Wang, Xueting Bai, Ganggang Shi

**Affiliations:** 1 Department of Pharmacology, Shantou University Medical College, Shantou, China; 2 Department of Pharmacy, Second Affiliated Hospital, Shantou University Medical College, Shantou, China; 3 Department of Biochemistry and Molecular Biology, Keck School of Medicine, University of Southern California, Los Angeles, California, United States of America; 4 Department of Chemistry, Shantou University Medical College, Shantou, China; 5 Department of Cardiovascular Diseases, First Affiliated Hospital, Shantou University Medical College, Shantou, China; Sun Yat-sen University Cancer Center, China

## Abstract

*Ipomoea stolonifera* (*I. stolonifera*) has been used for the treatment of inflammatory diseases including rheumatism and rheumatoid arthritis in Chinese traditional medicine. However, the anti-inflammatory activity of *I. stolonifera* has not been elucidated. For this reason, the anti-inflammatory activity of n-butanol extract of *I. stolonifera* (BE-IS) was evaluated *in vivo* by using acute models (croton oil-induced mouse ear edema, carrageenan-induced rat paw edema, and carrageenan-induced rat pleurisy) and chronic models (cotton pellet-induced rat granuloma, and complete Freund’s adjuvant (CFA)-induced rat arthritis). Results indicated that oral administration of BE-IS significantly attenuated croton oil-induced ear edema, decreased carrageenan-induced paw edema, reduced carrageenan-induced exudates and cellular migration, inhibited cotton pellet-induced granuloma formation and improved CFA-induced arthritis. Preliminary mechanism studies demonstrated that BE-IS decreased the levels of myeloperoxidase (MPO) and malondialdehyde (MDA), increased the activity of anti-oxidant enzyme superoxide dismutase (SOD) *in vivo*, and reduced the production of nitric oxide (NO), prostaglandin E_2_ (PGE_2_), tumor necrosis factor-α (TNF-α), interleukin (IL)-1β and IL-6 in lipopolysaccharide-activated RAW264.7 macrophages *in vitro*. Results obtained *in vivo* and *in vitro* demonstrate that BE-IS has considerable anti-inflammatory potential, which provided experimental evidences for the traditional application of *Ipomoea stolonifera* in inflammatory diseases.

## Introduction

The oceans have an abundance of bioactive natural products [1], many of which exhibit extraordinary chemical and structural features [2]–[4]. Likewise, there are special kinds of living organisms around the oceans, such as coastal plants [5], [6], some of which have pharmacological activities and therapeutic effects [7], [8]. For example, *Ipomoea pes-caprae*, a species in the *Convolvulaceae* family, is recognized to have anti-nociceptive and anti-inflammatory activity [9]–[11], and is used in Chinese traditional medicine to treat rheumatic diseases.

*Ipomoea stolonifera* (*I. stolonifera*), another coastal plant belonging to the same genus as *Ipomoea pes-caprae*, is believed to have more potent anti-inflammatory activity, and is distributed in tropical and sub-tropical regions, along dunes and coastal flats [12]. In the eastern area of Guangdong Province, China, it is widely used as a medicinal herb for disorders such as sunstroke, esocolitis and fish puncture wounds [13]. It also has been used to treat inflammatory disorders, such as rheumatism and arthritis. It is especially powerful in the treatment of rheumatoid arthritis.

Inflammation is the body’s immediate response of the immune system to infection and irritation [14]. It is like a double-edged sword because although inflammation eliminates invading pathogens and initiates the healing process, uncontrolled inflammation can lead to injury of tissues and cells, chronic inflammation, chronic diseases and neoplastic transformation [15]. At present, most inflammatory diseases are treated with conventional anti-inflammatory drugs, such as steroidal anti-inflammatory drugs (SAIDs) and nonsteroidal anti-inflammatory drugs (NSAIDs). However, prolonged use of these drugs may produce many adverse effects, including gastrointestinal disorders [16], [17], immunodeficiency and humoral disturbances [18]. We aimed to discover new anti-inflammatory agents with fewer adverse effects, and that could be developed for long-term administration.

Although *I. stolonifera* possesses remarkable curative effects, few studies have been conducted with the purpose of evaluating its pharmacological properties, including anti-inflammatory effects. For this reason, we investigated the anti-inflammatory activity of n-butanol extract from *I. stolonifera* (BE-IS).

It is well known that inflammation is a complex physiological response and may be acute and chronic [19]. Acute inflammation is to be activated when encountering harmful stimuli, which is characterized by not only vasodilatation, permeability accentuation and neutrophils infiltration, but also edema formation [20]. The acute inflammatory response will lead to chronic inflammation that is featured by tissue proliferation, granuloma, and repair [21].

In this study, we evaluated the anti-inflammatory effect of BE-IS using four *in vivo* animal models, that is, croton oil-induced mouse ear edema, carrageenan-induced rat paw edema, carrageenan-induced rat pleurisy and cotton pellet-induced rat granuloma, designed for examining the vasodilatation, edema formation, exudates and cellular migration, and tissue hyperplasia, respectively [21]. In addition, we used complete Freund’s adjuvant (CFA)-induced rat arthritis to evaluate the therapeutic effect of BE-IS for the treatment of arthritis.

Furthermore, in order to understand the preliminary anti-inflammatory mechanisms, we determined the levels of myeloperoxidase (MPO), superoxide dismutase (SOD) and malondialdehyde (MDA) *in vivo*, and measured the production of inflammatory mediators and cytokines (nitric oxide (NO), prostaglandin E_2_ (PGE_2_), tumor necrosis factor-α (TNF-α), interleukin (IL)-1β and IL-6) in lipopolysaccharide (LPS)-activated RAW264.7 macrophages *in vitro*.

## Materials and Methods

### Plant Material

Entire plants of *I. stolonifera*, including leaves, flowers, stems and roots were purchased at the Puning Folk Medicine Market in Shantou, China, which were collected at the beach of Chaoshan Area, Guangdong Province, China, and identified by Dr. Guangxiong Zhou (Department of Pharmacognosy, College of Pharmacy, Jinan University, Guangzhou, China). The plants were washed thoroughly with deionized water and dried in the shade at 30°C for 24 h. The voucher specimen of the plant material was deposited in the laboratory of Division of Traditional Chinese Medicine, Shantou University Medical College (SUMC), China.

### Chemicals and Reagents

Croton oil, carrageenan, CFA, 3-(4,5-dimethylthiazol-2-yl)-2,5-diphenyl tetrazolium bromide (MTT), dimethyl sulphoxide (DMSO) and LPS (Escherichia coli serotype 055:B5) were obtained from Sigma-Aldrich (St. Louis, MO, USA). MPO, SOD, MDA, total protein and NO kits were purchased from Nanjing Jiancheng Bioengineering Institute (Nanjing, China). PGE_2_ ELISA (enzyme-linked immunosorbent assay) kits were obtained from Enzo Life Sciences Inc. (NY, USA). TNF-α, IL-1β, and IL-6 ELISA kits were purchased from Boster Bioengineering Limited Company (Wuhan, China). All the other reagents were of analytical grade. Ultrapure water was used for the experiments.

### Animals and Ethics Statement

*In vivo* experiments were performed with male Kunming mice or male Sprague-Dawley rats kept under SPF (specific-pathogen free) conditions from the Laboratory Animal Center, SUMC. Animals were maintained in a room at a controlled temperature of 23±2°C and a 12-h light/dark cycle with free access to food and water. This animal study was approved by the Institutional Animal Care and Use Committee (IACUC) of SUMC, and all animal procedures were performed according to the IACUC policy. Animal studies including infection, intraperitoneal injection, and orbital venous bleeding of animals, were conducted in accordance with the recommendations in the Guidelines for the Care and Use of Laboratory Animals of the Ministry of Science and Technology of the People’s Republic of China ([2006]398).

### Preparation of Extracts from *I. stolonifera*

Dried *I. stolonifera* (1000 g) was ground to powder with a grinder, and extracted with 95% ethanol (5000 ml) for 3 days at 25°C, this procedure of which was repeated three times. After filtration and evaporation to dryness under reduced pressure at 50°C, the extract was decolored by light petroleum and exhaustively extracted with chloroform, ethyl acetate, n-butanol and water in turns, to obtain the respective extract. Of all the fractions obtained, the n-butanol extract of *I. stolonifera* (BE-IS) showed the strongest anti-inflammatory activity based on the carrageenan-induced paw edema test in rats. Therefore, we chose this extract to characterize anti-inflammatory activity. The dried BE-IS was dissolved in a solution of 0.5% sodium carboxymethyl cellulose (CMC-Na) for administration to test animals.

### Croton Oil-Induced Ear Edema in Mice and Assay for MPO Activity

Croton oil-induced ear edema was performed as described previously [22]–[24]. Mice (18∼22 g, 28-day-old) were divided randomly into the following five groups (20 mice per group) for oral gavage treatment once a day for 5 days: control group (0.5% CMC-Na, 0.2 ml); positive treatment group (300 mg/kg ibuprofen); and BE-IS groups (150, 300, and 600 mg/kg BE-IS). One hour after the last drug administration, ear edema was induced by topical application of 1% croton oil in mixture solvent (v/v, croton oil: ethanol: pyridine: ethyl ether = 1∶10∶20∶69, 100 ul) on the outer and inner surfaces of the right ear of each mouse. The left ear remained untreated and served as a control. An ear disk, 8.0 mm in diameter, was punched out and weighed 4 h after the application of the irritant. The weight difference between the left and the right ear disk of the same animal was evaluated as the extent of edema. The inhibition percentage was calculated by the following equation:
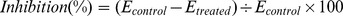
where E_control_ and E_treated_ is the extent of edema from the control group and treated groups.

Ear samples were collected and assayed for MPO activity [25], [26], which was performed according to the manufacturer’s instruction. The absorbance at 460 nm was measured using a microplate reader to determine the enzyme activity. The inhibition percentage was calculated as (1-MPO activity of the treated group/MPO activity of the control group) ×100%.

### Carrageenan-Induced Paw Edema in Rats

This rat model was performed as previously described [27]. Rats (130∼150 g, 35-day-old) were divided randomly into the following five groups (10 rats per group) for oral gavage treatment once a day for 7 days: control group (0.5% CMC-Na, 1 ml); positive treatment group (200 mg/kg ibuprofen); and BE-IS groups (100, 200, and 400 mg/kg BE-IS). One hour after the last administration, acute paw edema was induced by subplantar injection of 0.1 ml of 1% freshly prepared carrageenan suspension in normal saline into the right hind paw of each rat. Paw size was measured by wrapping a piece of cotton thread round the paw of each rat and recording the length of the thread, the paw circumference, by use of a metric ruler. Paws were measured immediately before and once an hour for 6 h after carrageenan injection. Inhibitory activity was calculated at 1–6 h after carrageenan injection with the following formula:
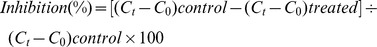
where C_t_ is paw size after carrageenan injection and C_0_ is paw size before carrageenan injection.

### Carrageenan-Induced Pleurisy in Rats

The method of Mikami and Miyasaka [28] was used. Rats (130∼150 g, 35-day-old) were divided randomly into five groups (10 rats per group) for the treatment once a day for 7 days: control group (0.5% CMC-Na, 1 ml, gavage); positive treatment group (2.5 mg/kg dexamethasone, i.p.); and BE-IS groups (100, 200, and 400 mg/kg BE-IS, gavage). One hour after the last administration of drug, rats were lightly anaesthetized under ether, and then 0.2 ml of normal saline or 1% carrageenan in normal saline was injected into the pleural cavity of each rat. 4 h after injection, rats were lightly anaesthetized, and blood samples were taken from the orbital pit. The serum was separated and stored at −20°C for measurement of MDA and SOD. Animals were then killed by an overdose of ether, and pleural cavities were exposed. The exudate volume was measured and the pleural cavity was washed with 2 ml ice-cold phosphate-buffered saline (pH 7.2) with heparin (5 U/ml). The exudates and washing were combined as the pleural exudates for the measurement of total protein. Exudates contaminated with blood were discarded. The total leukocyte number in the pleural exudates was counted in a Neubauer chamber.

### Cotton Pellet-Induced Granuloma in Rats

This experiment was performed as described [29] with slight modifications. A single needle incision was used to implant sterile cotton pellets, weighing 50±1 mg, into both axillae regions in rats (130∼150 g, 35-day-old) under ether anesthesia. Then rats were divided randomly into the following five groups (10 rats per group) for the treatment once a day for 7 days: control group (0.5% CMC-Na, 1 ml, gavage); positive treatment group (2.5 mg/kg dexamethasone, i.p.); and BE-IS groups (100, 200, and 400 mg/kg BE-IS, gavage). On day 8, granuloma tissue was carefully dissected. The pellets were incubated at 37°C for 24 h and dried at 60°C to constant weight. The increase in dry weight of the pellets was used to measure granuloma formation.

### CFA-Induced Chronic Arthritis in Rats

The rats (160∼180 g, 40-day-old) were divided randomly into the following five groups (10 rats per group) for oral gavage treatment once a day for 21 days: control group (0.5% CMC-Na, 1 ml); positive treatment group (200 mg/kg ibuprofen); and BE-IS groups (100, 200, and 400 mg/kg BE-IS). Arthritis was induced by intradermal injection of complete Freund’s adjuvant (0.1 ml) into the right hind paw [30]. The adjuvant contained heat-killed Mycobacterium tuberculosis (10 mg) in paraffin oil (1 ml). Swellings in the injected and contralateral hind paws were measured daily. The thickness of the paw was assessed by use of a vernier scale. The experimental and control groups were compared for differences in severity of arthritis.

### Acute Oral Toxicity – Fixed Dose Procedure

A fixed dose procedure was adopted to evaluate the acute oral toxicity of BE-IS according to Organization for Economic Co-operation and Development (OECD) Guidelines for the Testing of Chemicals [31]. Each animal, at the commencement of its dosing, was about 8 weeks old, and its weight fell within an interval of ±20% of the mean weight of any previously dosed mice. Groups of 10 mice were dosed in a stepwise procedure using the fixed doses of 750, 1500, 3000 and 6000 mg/kg. The initial dose level was selected on the basis of a sighting study as the dose expected to produce some signs of toxicity without causing severe toxic effects or mortality. Clinical signs and conditions associated with pain, suffering, and impending death, are described in detail in a separate OECD Guidance Document [32]. Additional groups of mice were dosed at higher or lower fixed doses, depending on the presence or absence of signs of toxicity or mortality. This procedure continues until the dose causing evident toxicity or no more than one death is identified, when no effects are seen at the highest dose or when deaths occur at the lowest dose.

### Cell Culture

The murine macrophage cell line, RAW264.7, was obtained from the Cell Bank, Chinese Academy of Science (Shanghai, China). Cells were cultured in Dulbecco’s modified Eagle’s medium (DMEM) (Gibco, USA) supplemented with 10% heat-inactivated fetal bovine serum (FBS) (HyClone, USA) and 1% penicillin (100 U/ml)-streptomycin (100 µg/ml) and incubated at 37°C in a humidified atmosphere with 5% CO_2_.

### Cell Viability Test

RAW264.7 cells (∼1×10^5^ cells/ml) were plated onto 96-well plates and incubated overnight. The cells were untreated or treated with 0.1% DMSO, 1 µg/ml LPS, various concentrations of BE-IS (1.25, 2.5, 5, 10, 20 µg/ml, dissolved in 0.1% DMSO) and then incubated at 37°C under a humidified atmosphere with 5% CO_2_ for 24 h. Cell viability was determined by MTT assay [33]. MTT (0.5 mg/ml) was added to each well, and cells were incubated for another 4 h. Then MTT was removed, and cells were lysed by addition of 150 µl/well DMSO. The optical density was measured at 550 nm on a microplate reader.

### Determination of NO, PGE_2_, TNF-α, IL-1β and IL-6

RAW264.7 cells (∼2×10^5^ cells/ml) were plated onto a 24-well plate and incubated at 37°C under a humidified atmosphere with 5% CO_2_ for 24 h. Then the cells were incubated with BE-IS (0, 1.25, 2.5, 5, 10, 20 µg/ml, dissolved in 0.1% DMSO) for 1 h before stimulation with LPS (1 µg/ml) for 24 h. The supernatants were collected and stored at −20°C before analysis. The concentrations of PGE_2_, TNF-α, IL-1β and IL-6 in the cell supernatants were determined by ELISA kits according to the manufacturers’ instructions. Nitrite accumulated in the culture medium was measured as an indicator of NO production based on the Griess reaction according to the manufacturer’s instructions.

### Statistical Analysis

Experimental results were expressed as mean ± standard error (S.E.). Individual group comparisons were analyzed by one-way analysis of variance (ANOVA), and the differences were calculated by using Tukey’s test (*P*<0.05) with SPSS 13.0 (SPSS Inc., Chicago, IL, USA). Values of *P*<0.05 were considered statistically significant.

## Results and Discussion

### Effect of BE-IS on Croton Oil-Induced Mouse Ear Edema and Tissue MPO Activity

**Figure 1:**
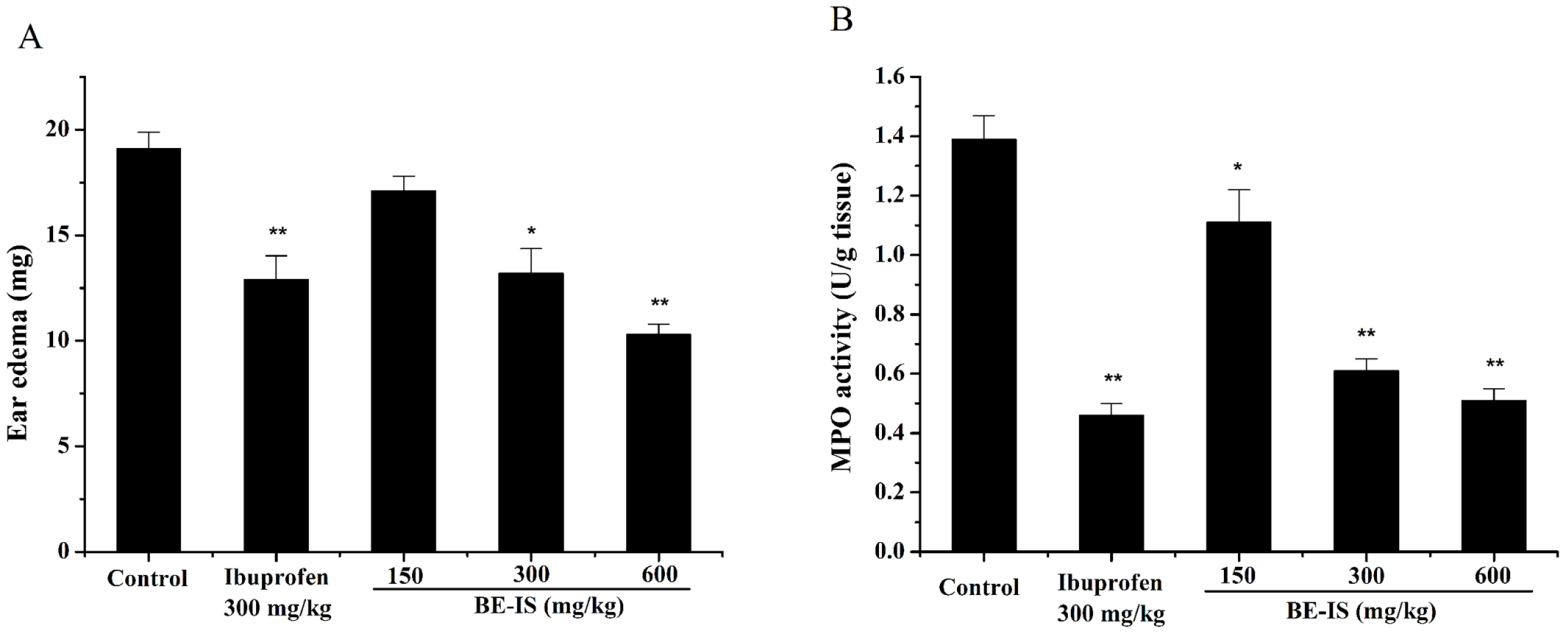
Effect of BE-IS on ear edema (A) and MPO activity (B) induced by croton oil in mice. Mice were divided randomly into five groups: Control group (0.5% CMC-Na), Ibuprofen group (300 mg/kg) and BE-IS groups (150, 300, and 600 mg/kg). Ear edema was induced by topical application of 1% croton oil on the outer and inner surfaces of the right ear of each mouse. The left ear remained untreated and served as a control. Ear edema and MPO activity were measured 4 h after application of croton oil. Values are expressed as mean ± S.E., n = 20, *P<0.05, **P<0.01 as compared to the control group.

Croton oil-induced ear edema is a widely used model for identifying potential anti-inflammatory agents [26]. Topical application of croton oil markedly elicited an inflammatory response in mice, as judged by edema formation and neutrophilic infiltration determined by the increase in the weight of the ear and in tissue MPO activity, respectively. BE-IS inhibited ear edema in a dose-dependent manner, with 10.3%, 30.1% and 45.8% inhibition occurring at doses of 150, 300 and 600 mg/kg, respectively (Figure 1A). At 600 mg/kg, BE-IS reduced ear edema to a greater extent than a 300 mg/kg dose of ibuprofen (*P*<0.01). These results indicate that BE-IS possesses anti-inflammatory activity against acute inflammation induced by croton oil. MPO is an enzyme present in the intracellular granules of neutrophils and is used as a marker for polymorphonuclear leukocyte infiltration into inflamed tissues, an indicator for inflammatory reaction [34]. Treatment with BE-IS at 150, 300 and 600 mg/kg, dose-dependently reduced MPO activity with a maximal effect of 63.3%. The reference drug ibuprofen (300 mg/kg) inhibited MPO activity by a similar 66.9% (Figure 1B).

### Effect of BE-IS on Carrageenan-Induced Rat Paw Edema

**Figure 2:**
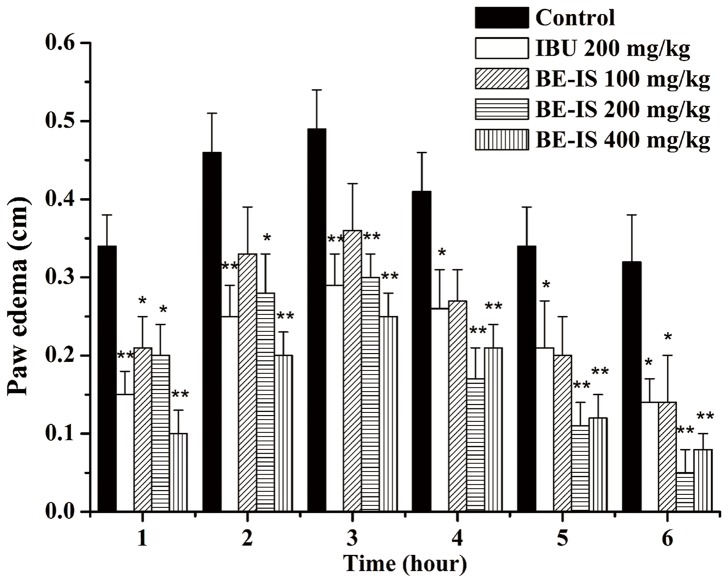
Effect of BE-IS on carrageenan-induced right hind paw edema in rats. Rats were divided randomly into five groups: Control group (0.5% CMC-Na), Ibuprofen group (200 mg/kg) and BE-IS groups (100, 200, and 400 mg/kg). Paw edema was induced by subplantar injection of 0.1 ml of 1% freshly prepared carrageenan suspension in normal saline into the right hind paw of each rat. Paw size was measured as the paw circumference immediately before and once an hour for 6 h after carrageenan injection. Values are expressed as mean ± S.E., n = 10, *P<0.05, ** <0.01 as compared to the control group.

To estimate the effect of BE-IS on acute inflammation, we used carrageenan-induced rat paw edema as an assay to reflect edema that occurs during the early stages of acute inflammation [35]. This model has been commonly employed to assess the anti-edematous effect of natural products [36]. The edematous response that occurs 0–2 h after carrageenan injection has been correlated with the exudative stage of inflammation, which is featured with the release of histamine, serotonin, and bradykinin [37]. The late phase, occurring about 3 h after carrageenan exposure when edema reaches its peak volume, is characterized by the presence of prostaglandins. Oral treatment with BE-IS significantly inhibited carrageenan-induced paw edema in rats. At a dose of 400 mg/kg, edema was remarkably suppressed at 1–3 h after carrageenan treatment, with inhibition being 70.1%, 56.5% and 49% at 1, 2, 3 h, respectively. At 4–6 h after carrageenan treatment, the 200 mg/kg BE-IS group exhibited the most potent inhibitory effect, with inhibition being 58.5%, 67.6%, 84.4% at 4, 5, 6 h, respectively. Ibuprofen (IBU) at 200 mg/kg inhibited paw edema induced by carrageenan significantly compared with the control group (*P*<0.01 at 1–3 h and *P*<0.05 at 4–6 h). The inhibition of BE-IS on each phase demonstrates that the extract has a non-selective inhibitory effect on the release or actions of those mediators mentioned before (Figure 2).

### Effect of BE-IS on Carrageenan-Induced Rat Pleurisy

One of the well-characterized acute inflammation models, carrageenan-induced pleurisy in rat, permits the quantitation and correlation of both exudates and cellular migration with changes in other inflammatory parameters [38]. Compared to the control group, oral pre-treatment with 400 mg/kg BE-IS significantly reduced the volume of pleural exudates and total leukocyte migration (*P*<0.01), inhibited the production of total protein in the pleural exudates (*P*<0.01), decreased the serum level of malondialdehyde (MDA) (*P*<0.05), an indicator of lipid peroxidation, and increased the serum level of superoxide dismutase (SOD) (*P*<0.01), an antioxidant enzyme. Dexamethasone (DEX), used as the reference drug, had a similar effect on total protein and SOD, but it is significantly more potent on exudate volume, leukocyte number and MDA (Table 1). The results showed that BE-IS had potent effect on increasing the serum level of SOD, which implies that BE-IS scavenges oxygen radicals by enhancing the activities of antioxidant enzymes. Thus, the anti-oxidative properties of BE-IS may contribute to the alleviation of the inflammatory response.

**Table 1:** Effect of BE-IS on rat pleurisy induced by carrageenan.

Group	Dose (mg/kg)	Exudate volume (ml)	Leukocyte number (10^3^/ul)	Protein (mg/ml)	MDA (nmol/ml)	SOD (U/ml)
Blank	–	0.03±0.01***	51.3±12***	19.95±1.29***	8.36±0.68**	254.72±8.02**
Control	–	1.77±0.12	119±14.6	35.03±3.37	13.74±1.16	204.01±8.69
DEX	2.5	0.63±0.13**	64.9±13.4***	25.32±4.51**	8.9±1.25**	225.72±9.73**
BE-IS	100	1.60±0.19**	114.8±20.6	29.23±3.44**	13.41±2.07	220.85±16.10**
BE-IS	200	1.13±0.17**	109.5±16.3	28.04±3.41**	12.95±2.43	225.22±16.76**
BE-IS	400	1.12±0.18**	82.4±19.7**	24.07±5.35**	12.27±1.19*	229.17±12.43**

Each value represents as mean ± S.E., n = 10.

*P<0.05,

**P<0.01,

***P<0.001 compared to control group.

### Effect of BE-IS on Cotton Pellet-Induced Rat Granuloma

**Figure 3:**
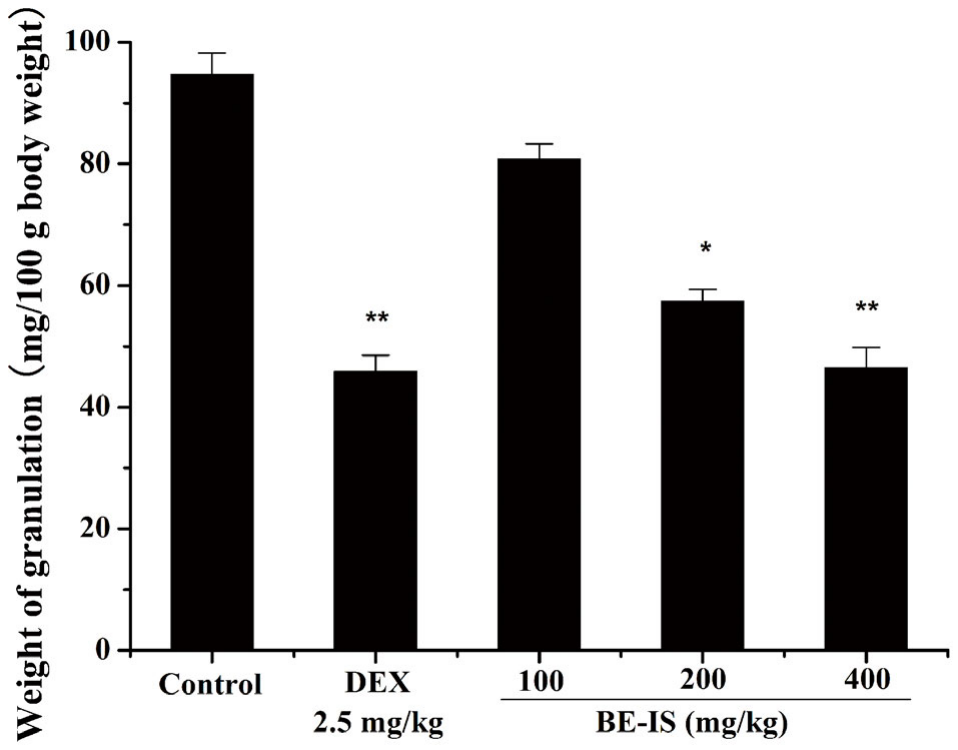
Effect of BE-IS on cotton pellet-induced granuloma in rats. With rats under ether anesthesia, sterile cotton pellets weighing 50±1 mg were implanted subcutaneously in both axillae regions of each rat by a single needle incision. Then rats were divided randomly into five groups: Control group (0.5% CMC-Na, p.o), Dexamethasone (DEX) group (2.5 mg/kg, i.p.) and BE-IS groups (100, 200, and 400 mg/kg, p.o). On day 8, granuloma tissue was carefully dissected. The pellets were incubated at 37°C for 24 h and dried at 60°C to constant weight. The increase in dry weight of the pellets was used to measure granuloma formation. Values are expressed as mean ± S.E., n = 10, *P<0.05, **P<0.01 as compared to the control group.

We used the rat cotton pellet-induced granuloma model for chronic inflammation to assess the effect of anti-inflammatory drugs on the proliferation phase of inflammation [39]. BE-IS at 200 and 400 mg/kg, as well as dexamethasone at 2.5 mg/kg, markedly inhibited granuloma formation surrounding cotton pellets compared with the vehicle control group. BE-IS reduced the formation of granuloma tissue in a dose-dependent manner. BE-IS at 400 mg/kg produced a maximal 50.8% inhibition, whereas 100 and 200 mg/kg produced 14.6% and 39.4% inhibition, respectively, as compared to 51.4% for dexamethasone (Figure 3). Thus, BE-IS can inhibit granuloma formation in the proliferation phase of the inflammatory process.

### Effect of BE-IS on CFA-Induced Rat Arthritis

CFA-induced chronic arthritis in rats is a convenient model for studying drugs affecting human arthritis. We administered BE-IS once daily to rats for 14 consecutive days and measured degree of swelling in treated and untreated hind paws. In adjuvant-induced arthritic animals, the soft swelling seen around the ankle joints is due to edema of periarticular tissues. Increased granulocytes and monocytes were associated with changes in ankle diameter [40]. BE-IS at 200 and 400 mg/kg gave potent inhibitory activity of chronic arthritis during the first 6 days, as evidenced by a significant reduction of paw edema in arthritic animals. Ibuprofen at 200 mg/kg inhibited paw edema induced by CFA to a similar extent as BE-IS at the dose of 400 mg/kg (Table 2).

**Table 2:** Effect of BE-IS on complete Freund’s adjuvant-induced chronic arthritic rats.

Day	Arthritic rat paw edema (mm)				
	Control	IBU(200 mg/kg)	BE-IS (100 mg/kg)	BE-IS (200 mg/kg)	BE-IS (400 mg/kg)
3	3.83±0.08	3.70±0.10*	3.76±0.12*	3.66±0.11**	3.58±0.17**
6	4.17±0.05	3.73±0.06**	4.09±0.10	3.95±0.06**	3.65±0.08**
9	3.54±0.08	3.43±0.05	3.52±0.11	3.53±0.10	3.43±0.05
12	3.43±0.04	3.44±0.05	3.37±0.09	3.39±0.06	3.44±0.05
21	4.14±0.18	3.77±0.14**	4.15±0.16	4.10±0.10	3.78±0.17**

Each value represents as mean ± S.E., n = 10.

*P<0.05,

**P<0.01 compared to control group.

### Acute Oral Toxicity of BE-IS in Mice

No mortality or severe toxic effects are seen even at the highest dose of 6000 mg/kg (Table 3). Hence, based on the OECD criteria, BE-IS is of relatively low acute toxicity hazard and its expected LD_50_ value exceeds 6000 mg/kg without the need for testing.

**Table 3:** Sighting study in acute oral toxicity assay–fixed dose procedure.

Dose (mg/kg)	The result of the sighting study
750	100% survival rate, no severe toxic effect*
1500	100% survival rate, no severe toxic effect*
3000	100% survival rate, no severe toxic effect*
6000	100% survival rate, no severe toxic effect*

*Clinical signs and conditions associated with pain, suffering, and impending death, are described in detail in a separate OECD Guidance Document [31].

### Cell Viability

**Figure 4:**
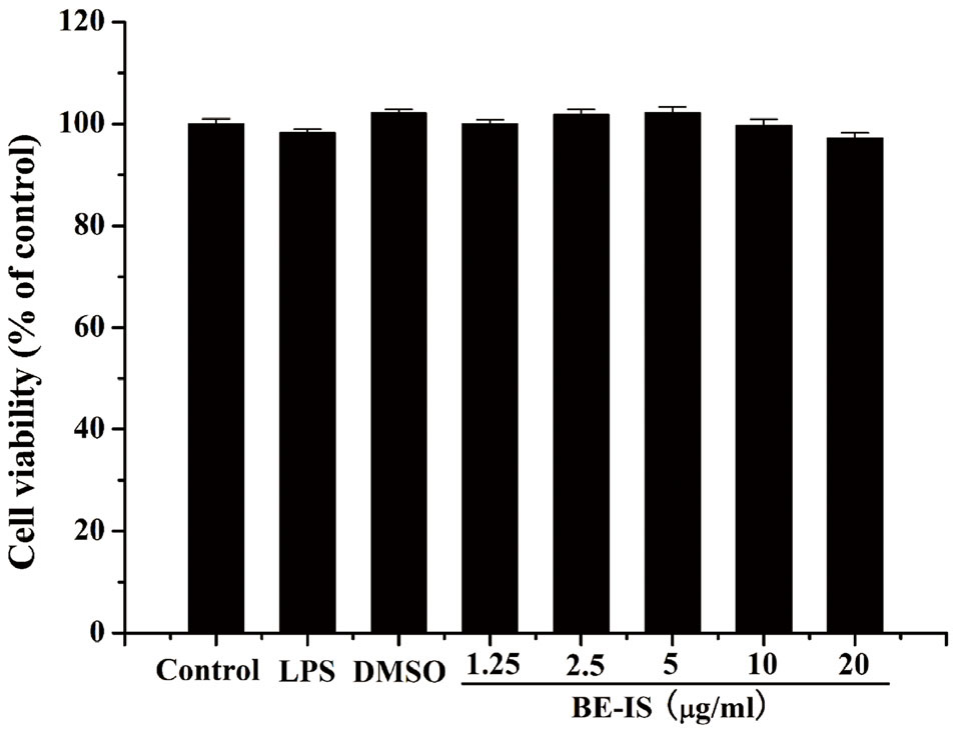
Effect of BE-IS on the viability of RAW264.7 cells. Cells were incubated for 24% DMSO, 1 µg/ml LPS, or BE-IS (1.25, 2.5, 5, 10, 20 µg/ml, dissolved in 0.1% DMSO). Cell viability was determined by MTT assay. The optical density was measured at 550 nm on a microplate reader. Values are expressed as mean ± S.E. for three different experiments performed in triplicate.

The potential cytotoxicity of BE-IS was evaluated by the MTT assay [33] after incubating cells for 24 h with 1.25, 2.5, 5, 10, or 20 µg/ml BE-IS to RAW264.7 cells. No cytotoxic effects were observed (Figure 4), suggesting that the anti-inflammatory effects of BE-IS are not due to a reduction in cell viability.

### Effect of BE-IS on the Production of Inflammatory Mediators

It is confirmed that RAW264.7 macrophages activated by LPS would generate massive inflammatory mediators and cytokines (e.g. NO, PGE_2_, TNF-α, IL-1β, IL-6), which would cause kinds of inflammatory diseases [41], [42]. Therefore, in this study, we chose LPS-activated RAW264.7 cell model to reveal anti-inflammatory mechanism of BE-IS.

**Figure 5:**
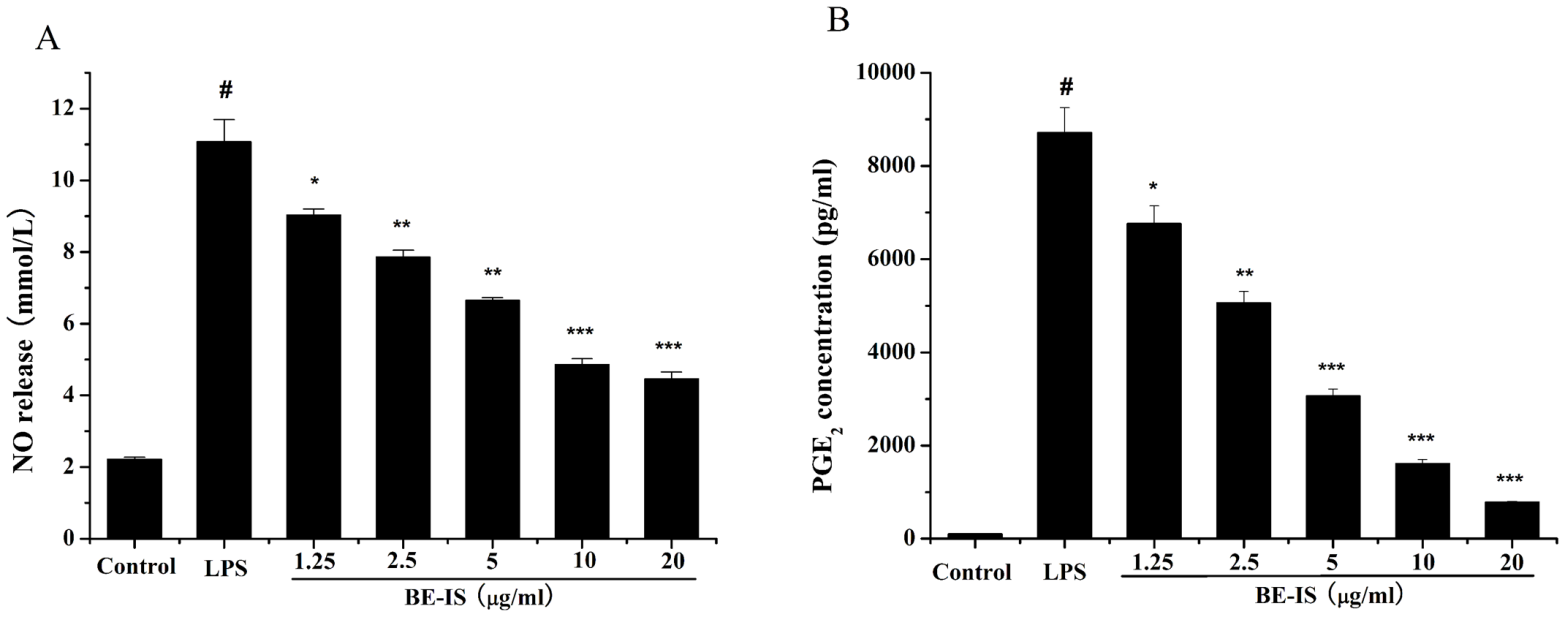
Effect of BE-IS on LPS-induced production of NO (A) and PGE_2_ (B) in RAW264.7 cells. Cells were incubated with BE-IS (0, 1.25, 2.5, 5, 10, 20 µg/ml) for 1 h before stimulation with LPS (1 µg/ml) for 24 h. The concentrations of NO and PGE_2_ in the cell supernatants were determined by the Griess reaction and ELISA, respectively, according to the manufacturers’ instructions. Values are expressed as mean ± S.E. for three independent experiments. ^#^ *P*<0.001 as compared to the control group, *P<0.05, **P<0.01, ***P<0.001 as compared to LPS-alone group.

The ability of BE-IS to modulate the production of inflammatory mediators NO and PGE_2_ was evaluated in LPS-induced RAW264.7 macrophages. NO and PGE_2_ levels in cell supernatants were respectively determined by the Griess assay and ELISA (Figures 5A and 5B). Treatment of RAW264.7 cells with LPS alone resulted in significant increases in both NO and PGE_2_ production as compared to that in the control group (*P*<0.001). However, BE-IS at 1.25–20 µg/ml markedly inhibited LPS-induced NO production in a dose-dependent manner. BE-IS was also found to significantly inhibit PGE_2_ production as compared to the LPS-treated group in a dose-dependent manner.

The pro-inflammatory mediators, NO and PGE_2_ produced by activated macrophages play critical roles in inflammatory diseases. NO is produced from L-arginine by nitric oxide synthetases and involved in various pathophysiological processes including inflammation [43]. At adequate concentrations, NO can generate or modify intracellular signals, thereby affecting the function of immune cells, as well as tumor cells and resident cells of different tissues and organs. However, its uncontrolled release can cause target tissue destruction during an infection [44]. PGE_2_ involved in inflammatory responses, is generated by the sequential metabolism of arachidonic acid by cycloxygenase (COX) [45]. Therefore, a drug capable of preventing the release of pro-inflammatory mediators in inflammatory cells could potentially possess anti-inflammatory activities. In this study, we found a non-cytotoxic dose range at which BE-IS inhibits the production of NO and PGE_2_ in LPS-induced RAW264.7 cells.

### Effect of BE-IS on the Production of Cytokines

**Figure 6:**
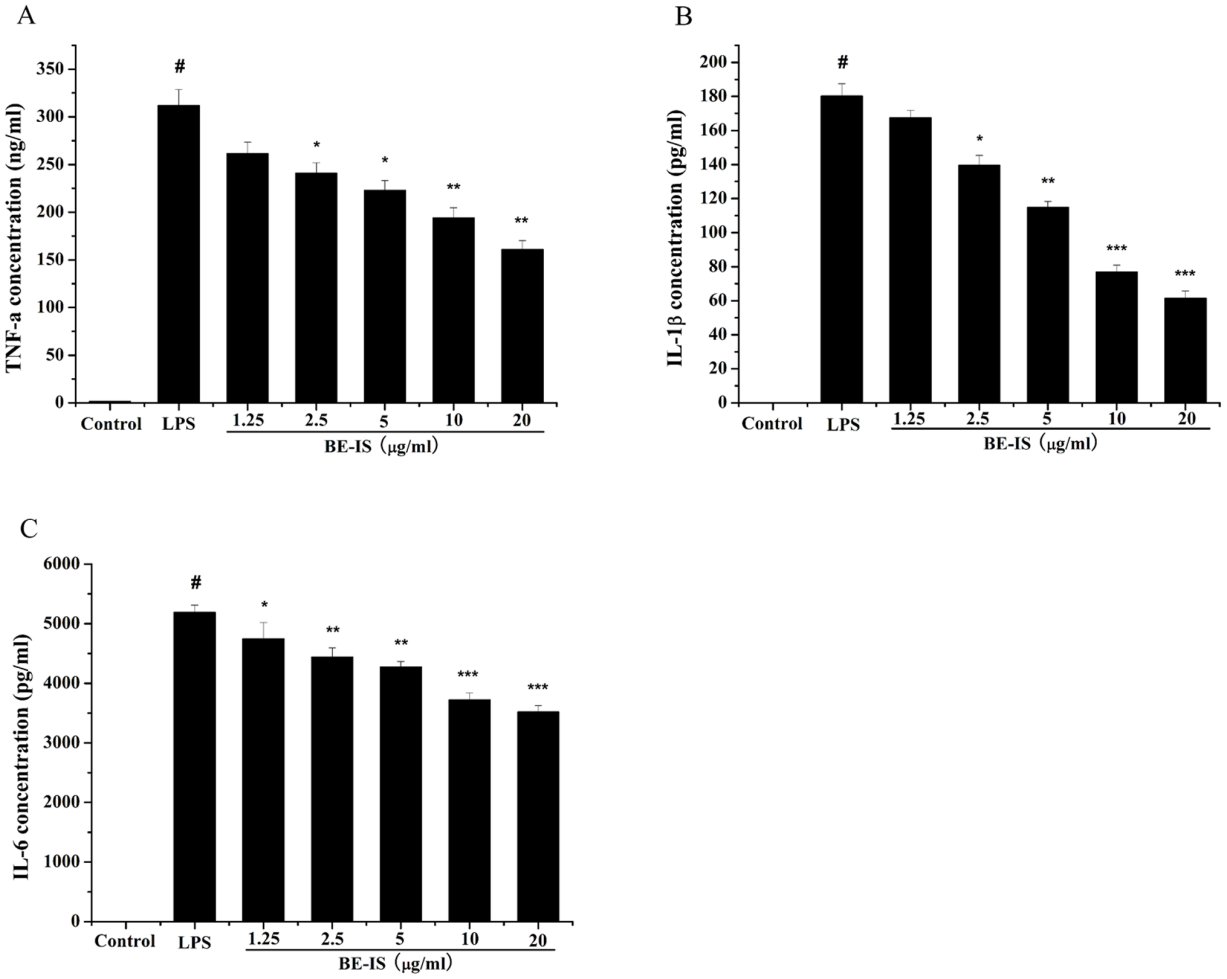
Effect of BE-IS on LPS-induced production of TNF-α (A), IL-1β (B) and IL-6 (C) in RAW264.7 cells. Cells were incubated with BE-IS (0, 1.25, 2.5, 5, 10, 20 µg/ml) for 1 h before stimulation with LPS (1 µg/ml) for 24 h. The concentrations of TNF-α, IL-1β and IL-6 in the cell supernatants were determined by ELISA according to the manufacturers’ instructions. Values are expressed as mean ± S.E. for three independent experiments. ^#^ *P*<0.001 as compared to the control group, *P<0.05, **P<0.01, ***P<0.001 as compared to LPS-alone group.

The concentrations of cytokines TNF-α, IL-1β and IL-6 in RAW264.7 cell supernatants were measured by ELISA (Figures 6A, 6B and 6C). Treatment of RAW264.7 cells with LPS alone resulted in a significant increase in cytokine production as compared to that in the control group (*P*<0.001). However, BE-IS at 2.5–20 µg/ml significantly decreased LPS-induced TNF-α (*P*<0.01 or *P*<0.05), IL-1β levels (*P*<0.001, *P*<0.01 or *P*<0.05), and IL-6 level (*P*<0.001, *P*<0.01 or *P*<0.05) levels in a dose-dependent manner.

Pro-inflammatory cytokines, such as TNF-α, IL-1β and IL-6 are known to contribute to tissue damage and multiple organ failure. They are considered to be important initiators of the inflammatory response and mediators of the development of various inflammatory diseases [46], [47]. TNF-α plays a key role in the induction and perpetuation of inflammation by activating macrophages and upregulating other pro-inflammatory cytokines and endothelial adhesion molecules [48]. Likewise, IL-1β is one of the most important inflammatory cytokines secreted by macrophages, and is induced by LPS. During inflammation, increases in the release of IL-1β lead to cell or tissue damage [49], [50]. IL-6 is also a pivotal pro-inflammatory cytokine, regarded as an endogenous mediator of LPS-induced fever [51]. In the present study, we demonstrate that BE-IS inhibits TNF-α, IL-1β and IL-6 production in a dose-dependent manner in LPS-stimulated RAW264.7 cells.

## Conclusions

In the present study, we investigated the anti-inflammatory activity of BE-IS *in vivo* using acute and chronic inflammatory animal models, and *in vitro* using LPS-activated RAW264.7 cells. We show that treatment with BE-IS inhibits the inflammatory response, as judged by decrease in croton oil-induced ear edema and carrageenan-induced paw edema, reduction of carrageenan-induced exudates and cellular migration, inhibition of cotton pellet-induced granuloma formation, and amelioration of CFA-induced arthritis. Preliminary mechanism studies demonstrated that BE-IS decreased the levels of MPO and MDA, increased the activity of anti-oxidant enzyme SOD *in vivo*, and reduced the production of NO, PGE_2_, TNF-α, IL-1β and IL-6 in LPS-activated RAW264.7 cells *in vitro*. These results suggest that *I. stolonifera* possesses considerable anti-inflammatory activity, and may become a potential source of natural anti-inflammatory agents. Therefore, in our next work, we will focus on the study of chemical constituents of *I. stolonifera*.

## References

[1] Mondol MA, Shin HJ, Islam MT (2013) Diversity of secondary metabolites from marine Bacillus species: chemistry and biological activity. Mar Drugs 11: 2846–2872. 23941823 10.3390/md11082846 PMC3766869

[2] Costantino V, Fattorusso E, Menna M, Taglialatela-Scafati O (2004) Chemical diversity of bioactive marine natural products: an illustrative case study. Curr Med Chem 11: 1671–1692. 15279576 10.2174/0929867043364973

[3] Hou Y, Harinantenaina L (2010) New and bioactive natural products isolated from madagascar plants and marine organisms. Curr Med Chem 17: 1191–1219. 20158472 10.2174/092986710790827834

[4] Nasri R, Nasri M (2013) Marine-derived bioactive peptides as new anticoagulant agents: a review. Curr Protein Pept Sci 14: 199–204. 23721316 10.2174/13892037113149990042

[5] Odum WE (1977) Coastal plants. Science 197: 1071–1072. 10.1126/science.197.4308.1071-a 17836071

[6] Matallana G, Wendt T, Araujo DS, Scarano FR (2005) High abundance of dioecious plants in a tropical coastal vegetation. Am J Bot 92: 1513–1519. 21646169 10.3732/ajb.92.9.1513

[7] Ravikumar S, Nazar S, Nuralshiefa A, Abideen S (2005) Antibacterial activity of traditional therapeutic coastal medicinal plants against some pathogens. J Environ Biol 26: 383–386. 16334271

[8] Inbaneson SJ, Ravikumar S, Suganthi P (2012) In vitro antiplasmodial effect of ethanolic extracts of coastal medicinal plants along Palk Strait against Plasmodium falciparum. Asian Pac J Trop Biomed 2: 364–367. 23569931 10.1016/S2221-1691(12)60057-4 PMC3609306

[9] de Souza MM, Madeira A, Berti C, Krogh R, Yunes RA, et al (2000) Antinociceptive properties of the methanolic extract obtained from Ipomoea pes-caprae (L.) R. Br. J Ethnopharmacol 69: 85–90. 10661888 10.1016/s0378-8741(99)00142-7

[10] Krogh R, Kroth R, Berti C, Madeira AO, Souza MM, et al (1999) Isolation and identification of compounds with antinociceptive action from Ipomoea pes-caprae (L.) R. Br. Pharmazie 54: 464–466. 10399194

[11] Pongprayoon U, Baeckstrom P, Jacobsson U, Lindstrom M, Bohlin L (1991) Compounds inhibiting prostaglandin synthesis isolated from Ipomoea pes-caprae. Planta Med 57: 515–518. 1818340 10.1055/s-2006-960196

[12] Williams A, Feagin R (2010) Sargassum as a natural solution to enhance dune plant growth. Environ Manage 46: 738–747. 20859628 10.1007/s00267-010-9558-3

[13] Qiu H (1995) Notes relating to the flora of southern China (3). Guihaia 15: 7–12.

[14] Ialenti A, Moncada S, Di Rosa M (1993) Modulation of adjuvant arthritis by endogenous nitric oxide. Br J Pharmacol 110: 701–706. 8242242 10.1111/j.1476-5381.1993.tb13868.x PMC2175935

[15] Mantovani A, Allavena P, Sica A, Balkwill F (2008) Cancer-related inflammation. Nature 454: 436–444. 18650914 10.1038/nature07205

[16] Roth SH (2012) Coming to terms with nonsteroidal anti-inflammatory drug gastropathy. Drugs 72: 873–879. 22564130 10.2165/11633740-000000000-00000

[17] Nagatomi H, Ando K (1984) Studies on the anti-inflammatory activity and ulcerogenic adverse effect of thiazole derivatives, especially 2-amino-thiazoleacetic acid derivatives. Arzneimittelforschung 34: 599–603. 6540578

[18] Simon RA (2003) Prevention and treatment of reactions to NSAIDs. Clin Rev Allergy Immunol 24: 189–198. 12668898 10.1385/CRIAI:24:2:189

[19] Medzhitov R (2008) Origin and physiological roles of inflammation. Nature 454: 428–435. 18650913 10.1038/nature07201

[20] Huang MH, Wang BS, Chiu CS, Amagaya S, Hsieh WT, et al (2011) Antioxidant, antinociceptive, and anti-inflammatory activities of Xanthii Fructus extract. J Ethnopharmacol 135: 545–552. 21466841 10.1016/j.jep.2011.03.057

[21] Eddouks M, Chattopadhyay D, Zeggwagh NA (2012) Animal models as tools to investigate antidiabetic and anti-inflammatory plants. Evid Based Complement Alternat Med 2012: 142087. 22899950 10.1155/2012/142087 PMC3414199

[22] Tubaro A, Dri P, Delbello G, Zilli C, Della Loggia R (1986) The croton oil ear test revisited. Agents Actions 17: 347–349. 3962781 10.1007/BF01982641

[23] Tubaro A, Dri P, Melato M, Mulas G, Bianchi P, et al (1986) In the croton oil ear test the effects of non steroidal antiinflammatory drug (NSAIDs) are dependent on the dose of the irritant. Agents Actions 19: 371–373. 3825756 10.1007/BF01971259

[24] Morteza-Semnani K, Saeedi M, Hamidian M (2004) Anti-inflammatory and analgesic activity of the topical preparation of Glaucium grandiflorum. Fitoterapia 75: 123–129. 15030915 10.1016/j.fitote.2003.12.007

[25] Bradley PP, Priebat DA, Christensen RD, Rothstein G (1982) Measurement of cutaneous inflammation: estimation of neutrophil content with an enzyme marker. J Invest Dermatol 78: 206–209. 6276474 10.1111/1523-1747.ep12506462

[26] De Young LM, Kheifets JB, Ballaron SJ, Young JM (1989) Edema and cell infiltration in the phorbol ester-treated mouse ear are temporally separate and can be differentially modulated by pharmacologic agents. Agents Actions 26: 335–341. 2567568 10.1007/BF01967298

[27] Winter CA, Risley EA, Nuss GW (1962) Carrageenin-induced edema in hind paw of the rat as an assay for antiiflammatory drugs. Proc Soc Exp Biol Med 111: 544–547. 14001233 10.3181/00379727-111-27849

[28] Mikami T, Miyasaka K (1983) Effects of several anti-inflammatory drugs on the various parameters involved in the inflammatory response in rat carrageenin-induced pleurisy. Eur J Pharmacol 95: 1–12. 6583058 10.1016/0014-2999(83)90261-3

[29] Winter CA, Porter CC (1957) Effect of alterations in side chain upon anti-inflammatory and liver glycogen activities of hydrocortisone esters. J Am Pharm Assoc Am Pharm Assoc (Baltim) 46: 515–519. 10.1002/jps.3030460902 13491439

[30] Ahmad SF, Khan B, Bani S, Suri KA, Satti NK, et al (2006) Amelioration of adjuvant-induced arthritis by ursolic acid through altered Th1/Th2 cytokine production. Pharmacol Res 53: 233–240. 16406805 10.1016/j.phrs.2005.11.005

[31] OECD Guidance Document on the Recognition, Assessment and Use of Clinical Signs as Humane Endpoints for Experimental Animals Used in Safety Evaluation (2000) OECD Publishing.

[32] OECD Guidance Document on Acute Oral Toxicity Testing (2002) OECD Publishing.

[33] Mosmann T (1983) Rapid colorimetric assay for cellular growth and survival: application to proliferation and cytotoxicity assays. J Immunol Methods 65: 55–63. 6606682 10.1016/0022-1759(83)90303-4

[34] Cabrini DA, Moresco HH, Imazu P, da Silva CD, Pietrovski EF, et al (2011) Analysis of the Potential Topical Anti-Inflammatory Activity of Averrhoa carambola L. in Mice. Evid Based Complement Alternat Med 2011: 908059. 21785638 10.1093/ecam/neq026 PMC3137785

[35] Matsuda R, Tanihata S (1992) [Suppressive effect of sialic acid on the prostaglandin E2-mediated edema in carrageenin-induced inflammation of rat hind paws]. Nihon Yakurigaku Zasshi 99: 363–372. 1592319 10.1254/fpj.99.363

[36] Kang M, Jung I, Hur J, Kim SH, Lee JH, et al (2010) The analgesic and anti-inflammatory effect of WIN-34B, a new herbal formula for osteoarthritis composed of Lonicera japonica Thunb and Anemarrhena asphodeloides BUNGE in vivo. J Ethnopharmacol 131: 485–496. 20643199 10.1016/j.jep.2010.07.025

[37] Antonisamy P, Duraipandiyan V, Ignacimuthu S (2011) Anti-inflammatory, analgesic and antipyretic effects of friedelin isolated from Azima tetracantha Lam. in mouse and rat models. J Pharm Pharmacol 63: 1070–1077. 21718291 10.1111/j.2042-7158.2011.01300.x

[38] Vinegar R, Truax JF, Selph JL, Voelker FA (1982) Pathway of onset, development, and decay of carrageenan pleurisy in the rat. Fed Proc 41: 2588–2595. 6806127

[39] Selye H (1953) On the mechanism through which hydrocortisone affects the resistance of tissues to injury; an experimental study with the granuloma pouch technique. J Am Med Assoc 152: 1207–1213. 13061241 10.1001/jama.1953.63690130001006

[40] Rasool M, Varalakshmi P (2007) Protective effect of Withania somnifera root powder in relation to lipid peroxidation, antioxidant status, glycoproteins and bone collagen on adjuvant-induced arthritis in rats. Fundam Clin Pharmacol 21: 157–164. 17391288 10.1111/j.1472-8206.2006.00461.x

[41] Barbour SE, Wong C, Rabah D, Kapur A, Carter AD (1998) Mature macrophage cell lines exhibit variable responses to LPS. Molecular Immunology 35: 977–987. 9881693 10.1016/s0161-5890(98)00070-4

[42] Park HH, Kim MJ, Li Y, Park YN, Lee J, et al (2013) Britanin suppresses LPS-induced nitric oxide, PGE2 and cytokine production via NF-kappaB and MAPK inactivation in RAW 264.7 cells. Int Immunopharmacol 15: 296–302. 23270759 10.1016/j.intimp.2012.12.005

[43] Raetz CR (1993) Bacterial endotoxins: extraordinary lipids that activate eucaryotic signal transduction. J Bacteriol 175: 5745–5753. 8376321 10.1128/jb.175.18.5745-5753.1993 PMC206651

[44] Kim HW, Murakami A, Williams MV, Ohigashi H (2003) Mutagenicity of reactive oxygen and nitrogen species as detected by co-culture of activated inflammatory leukocytes and AS52 cells. Carcinogenesis 24: 235–241. 12584172 10.1093/carcin/24.2.235

[45] Vane JR, Bakhle YS, Botting RM (1998) Cyclooxygenases 1 and 2. Annu Rev Pharmacol Toxicol 38: 97–120. 9597150 10.1146/annurev.pharmtox.38.1.97

[46] Glauser MP (1996) The inflammatory cytokines. New developments in the pathophysiology and treatment of septic shock. Drugs 52 Suppl 29–17. 10.2165/00003495-199600522-00004 8869831

[47] Mannel DN, Echtenacher B (2000) TNF in the inflammatory response. Chem Immunol 74: 141–161. 10608086

[48] Beutler B, Cerami A (1989) The biology of cachectin/TNF–a primary mediator of the host response. Annu Rev Immunol 7: 625–655. 2540776 10.1146/annurev.iy.07.040189.003205

[49] Molloy RG, Mannick JA, Rodrick ML (1993) Cytokines, sepsis and immunomodulation. Br J Surg 80: 289–297. 8472134 10.1002/bjs.1800800308

[50] West MA, Bennet T, Clair L (1995) Reprogrammed macrophage tumor necrosis factor and interleukin-1 release with inflammatory pretreatment: differential regulation by endotoxin and zymosan. J Trauma 39: 404–410. 7473900 10.1097/00005373-199509000-00002

[51] Van Snick J (1990) Interleukin-6: an overview. Annu Rev Immunol 8: 253–278. 2188664 10.1146/annurev.iy.08.040190.001345

